# Cognitive Frailty and Functional Disability Among Community-Dwelling Older Adults: A Systematic Review

**DOI:** 10.1093/geroni/igad005

**Published:** 2023-01-23

**Authors:** Kar Foong Tang, Pei-Lee Teh, Shaun Wen Huey Lee

**Affiliations:** School of Pharmacy, Monash University Malaysia, Selangor, Malaysia; School of Business, Monash University Malaysia, Selangor, Malaysia; Gerontechnology Laboratory, Monash University Malaysia, Selangor, Malaysia; School of Pharmacy, Monash University Malaysia, Selangor, Malaysia; Gerontechnology Laboratory, Monash University Malaysia, Selangor, Malaysia

**Keywords:** Activities of daily living, Adverse health outcome, Mobility

## Abstract

**Background and Objectives:**

This review aimed to summarize the association between cognitive frailty (presence of frailty and cognitive impairment) and the risk of disabilities in activities of daily living (ADL), instrumental ADL (IADL), mobility, or other functional disabilities among older adults.

**Research Design and Methods:**

PubMed, Embase, CINAHL Plus, and PsycINFO were searched from January 2001 to May 14, 2022, for observational studies that reported cognitive frailty among community-dwelling individuals aged 60 years and above. Results were narratively synthesized.

**Results:**

Eleven studies encompassing 44 798 participants were included, with a prevalence of cognitive frailty ranging from 1.4% to 39.3%. Individuals with cognitive frailty were more likely to develop disabilities in ADL and IADL compared to robust (absence of frailty and cognitive impairment) individuals. Significant disability burden and elevated risk of combined ADL/IADL disability or physical limitation among participants with cognitive frailty were reported. There was limited evidence on the association between cognitive frailty and mobility disability.

**Discussion and Implications:**

Individuals with cognitive frailty were likely at higher risk of developing functional disability and incurring higher disability burden than robust individuals, but evidence remains limited for those with prefrailty with cognitive impairment. Further research on this gap and standardization of cognitive frailty assessments would facilitate comparisons across populations.

**PROSPERO Registration:**

CRD42021232222

**Translational Significance:** Physical frailty and cognitive impairment are each independently associated with the risk of disability in activities of daily living (ADL) and instrumental ADL (IADL). However, gaps remain in understanding the risk of functional disability in the presence of concurrent physical frailty and cognitive impairment (cognitive frailty). This review showed that cognitive frailty is associated with an increased risk of ADL and IADL disabilities, but evidence is limited for mobility disability. This knowledge would be useful in determining disability burden, supporting interventions to delay cognitive frailty and functional disability, and early detection of frailty and cognitive impairment among older adults.

## Background and Objectives

Frailty is a state of increased vulnerability due to the decline in the function of various physiologic systems (1,2). This increases an older adult’s risk of developing adverse health outcomes (3–5) and represents a public health concern in light of the global aging population. However, to date, frailty remains a dynamic yet heterogeneous concept due to a lack of consensus on its definition (6–8). The most commonly used construct is the frailty phenotype proposed by Fried and colleagues, which is characterized by shrinking (unintentional weight loss), weakness, and poor endurance as indicated by exhaustion, slowness, and low physical activity level (1). Individuals without these characteristics are considered robust, those with 1 or 2 characteristics are considered prefrail, and those with 3 or more characteristics are considered frail.

The construct primarily focuses on the physical domain of frailty, but some studies suggest that frailty represents a multidimensional concept and that the cognitive domain should be included in the definition of frailty (9,10), as both domains are thought to be interlinked (7,8,11). As such, the concept of cognitive frailty was introduced to encompass a broader definition of frailty, where an older adult exhibits the simultaneous presence of physical frailty and cognitive impairment in the absence of dementia (10). The International Academy on Nutrition and Aging and the International Association of Gerontology and Geriatrics were the first international consensus group to provide this definition of cognitive frailty in older adults in 2013 (10) to conceptualize cognitive impairment due to physical frailty and not the presence of neurological condition. The definition of cognitive impairment is heterogeneous, ranging from impairment in 1 or more cognitive domains, cognitive changes reported by the patient, caregiver or physician, or impairment based on clinical dementia rating (CDR). The consensus group suggested using a comprehensive cognitive and physical frailty assessment to identify cognitive frailty (physical frailty and cognitive impairment).

Physical frailty has been widely reported to be predictive of adverse health outcomes, ranging from mortality, hospitalization, falls, fractures, disability, cognitive decline, and institutionalization (12–14). Studies on the association between physical frailty and disability have indicated that older adults with physical frailty had higher odds of developing disability than robust older adults, although this association was not found in some studies (14,15). In comparison, evidence on the association between cognitive frailty and adverse health outcomes other than mortality, such as functional disability, is still relatively underexplored (16). In a previous systematic review on adverse outcomes of cognitive frailty (16), 2 studies identified in the review reported on the relationship between cognitive frailty and activities of daily living (ADL) or instrumental ADL (IADL) dependency, which showed a two- to fivefold increased risk of dependence. Another review (17) reported 6 other studies which evaluated the association between cognitive frailty and disability. Of the additional 6 studies, only 1 reported on mobility disability, but the association could not be determined because all 4 participants with cognitive frailty developed mobility disability during the study follow-up period (18). In these 2 reviews, the authors found only a limited number of studies reporting disability as an adverse outcome of cognitive frailty whereas 2 other reviews (19,20) did not specify the types of disability measured. These reflect large gaps in cognitive frailty studies, which have mostly focused on well-known adverse outcomes and less on disability due to challenges in measuring and comparing disability, especially mobility disability, which represents an important health indicator for older adults.

In line with a rapidly aging population globally, an increasing proportion of older adults will have declining functional status and an increased risk of frailty and cognitive impairment, among other age-related conditions. There remains a gap in understanding how frailty and cognitive impairment affect functional ability, such as ADL or IADL, among older adults because frailty and cognitive impairment have usually been studied separately. Physical frailty (14) and cognitive impairment (21) are known independent risk factors of ADL or IADL disability, but it is unclear if the concurrent presence of both frailty and cognitive impairment will accentuate the risk of developing functional disability. Frailty (22,23), cognitive impairment, and functional disability (24) are expected to contribute to increased healthcare burden and cost due to the increased need for health and social care services, whereas the increase in cognitive frailty prevalence over time (25) is also likely to increase adverse outcomes among older adults.

Given the implications of frailty on health outcomes and healthcare utilization (8,22), there is a need for a focused review to understand the occurrence of functional disabilities in the presence of physical frailty and cognitive impairment (cognitive frailty). This can help guide frailty prevention strategies and interventions to maintain functional ability in older adults. This review aims to summarize the association between cognitive frailty and the risk of developing functional disability among community-dwelling older adults. In this review, we included functional disabilities, namely ADL, IADL, and mobility disabilities; limitations in physical function; and other functional disabilities to provide readers with an overview of the association between cognitive frailty and risk of functional disability.

## Research Design and Methods

The systematic review protocol was registered in PROSPERO (CRD42021232222). Reporting was conducted according to the Preferred Reporting Items for Systematic Reviews and Meta-Analyses (PRISMA) guidelines (26).

### Search Strategy

A systematic search was conducted in four databases: PubMed, EMBASE, CINAHL Plus, and PsycINFO from 2001 (corresponding to the year when the Fried frailty phenotype construct was published) until May 14, 2022. The search included a combination of keywords and Medical Subject Headings terms related to frailty, older adults, ADL, disability, mobility, physical function, and adverse outcome. The list of databases and search terms are available in Supplementary Material. Reference lists of included articles and previous systematic reviews on adverse outcomes of cognitive frailty were screened to identify additional studies.

### Selection Criteria

Observational studies (cross-sectional or longitudinal cohort studies) were included if they reported on community-dwelling older adults aged 60 years and above. This age cutoff point was selected because studies on frailty typically included participants aged 60 years and above (27). Cross-sectional, prospective cohort studies were included due to the small number of longitudinal studies that reported on the association between cognitive frailty and disability. Cognitive frailty was defined by the presence of frailty or prefrailty, and concurrent cognitive impairment was identified using validated physical frailty and cognitive assessments. A preliminary literature search has identified that most studies on cognitive frailty have slightly modified the definition of cognitive frailty by the consensus group, defining this condition with the presence of mild cognitive impairment instead of a CDR of 0.5 with the exclusion of concurrent Alzheimer’s disease or other dementias, and physical frailty using the modified Fried frailty phenotype (28). Thus, the utilization of CDR was not compulsory for study inclusion in this review if a validated cognitive assessment tool was reported. Studies must report the association between cognitive frailty and functional disability (ADL or IADL, mobility, physical function).

Studies were excluded if they included hospitalized or institutionalized older adults or those with neurological disorders or dementia. Conference abstracts, reviews, randomized controlled trials, protocols, and studies published in other languages besides English were excluded. Study titles and abstracts were screened based on the inclusion/exclusion criteria, and full texts of relevant studies were screened for eligibility. Data were extracted using a piloted data extraction form, including study and participant characteristics, frailty assessment and classification, and corresponding disability outcomes and measurement. Data extraction was conducted by 1 reviewer (K.F.T.) and checked by the second reviewer (S.W.H.L.), with discrepancies resolved by consensus.

### Quality Assessment

The methodological quality of included studies was evaluated using the Joanna Briggs Institute’s Critical Appraisal Checklist for cohort and cross-sectional studies. Two reviewers independently conducted the assessment, with disagreements resolved by consensus.

### Data Analysis

The primary outcomes were ADL, IADL, and mobility disabilities. Other outcomes include the combination of disabilities and physical function limitations. Data were synthesized narratively and summarized using a Harvest plot (29,30) due to the small number of studies, heterogeneous outcome measurement, and cognitive frailty definition. Findings were reported based on fully adjusted statistical measures with a 95% confidence interval (CI). In the reporting of results, participants were categorized into 4 groups defined previously: (a) robust (absence of frailty and cognitive impairment), (b) prefrailty with cognitive impairment, (c) cognitive frailty (physical frailty and cognitive impairment), and (d) combined cognitive frailty (only for studies which grouped both frail and prefrail participants with cognitive impairment). Comparison between groups was made by using the robust group as the reference.

## Results

### Study Characteristics

A total of 18 184 records were screened, 170 studies were selected for full-text screening, and 11 records were included in this review (Supplementary Figure 1). The 11 studies included 44 798 participants, with mean age ranging from 67.7 to 75.2 years. These include 9 cohort studies with a follow-up duration of between 1 and 11 years (3,18,31–37) and 2 cross-sectional studies(Supplementary Table 1) (38,39). Nine studies were conducted in high-income countries, and 2 were in upper–middle-income countries. No studies were identified from low- and lower–middle-income countries.

### Definition of Frailty and Cognitive Frailty by Studies

Physical frailty was evaluated using the Fried frailty phenotype (1) or its modified version in all studies except for one that used walking and grip strength measurements (39). Older adults were classified as physically frail in the presence of 3 or more phenotype criteria, prefrail in the presence of 1 or 2 phenotype criteria, and robust in the absence of phenotype criteria. Cognitive function was most commonly assessed using the Mini-Mental State Examination (31,33,36–38). Several studies used other measures, such as the National Center for Geriatrics and Gerontology–Functional Assessment Tool (Supplementary Table 1). The definition of cognition function/impairment varied across studies as classifications were based on cognitive test score distributions or cutoff points set for the population. The prevalence of cognitive frailty reported in 11 studies varied widely, ranging from 1.4% to 39.3% due to differences in cognitive frailty classification.

ADL disability was reported in 5 studies (3,18,31,32,37), IADL disability in 4 studies (18,31,37,39), and mobility disability in 3 studies (18,31,37). Participants were classified as having a disability if they were free from disability at baseline but could not perform at least 1 activity without assistance during follow-up assessment. Other functional disabilities and physical function limitations were reported in 4 studies (33–36) and are summarized in Supplementary Table 2.

### Quality Assessment

The methodological quality assessment of studies is shown in Supplementary Table 3. All studies were judged to be representative based on sampling methods but may be biased due to recruitment procedures (e.g., recruitment at community health centers). All studies reported covariate adjustments due to confounding such as age and gender. Two studies were assessed to have an inadequate follow-up of cohorts due to attrition (33,35). The highest risk of bias was present in outcome assessment, given that disability was based on self-reporting by participants during interviews, and there would be a possible risk of recall bias, especially among participants with cognitive impairment. All studies have excluded participants with functional disability at baseline, but 7 studies did not have exclusion criteria for participants with Parkinson’s disease, which is known to increase the risk of functional disability.

### Primary Outcomes

#### ADL disability

**Figure 1.**
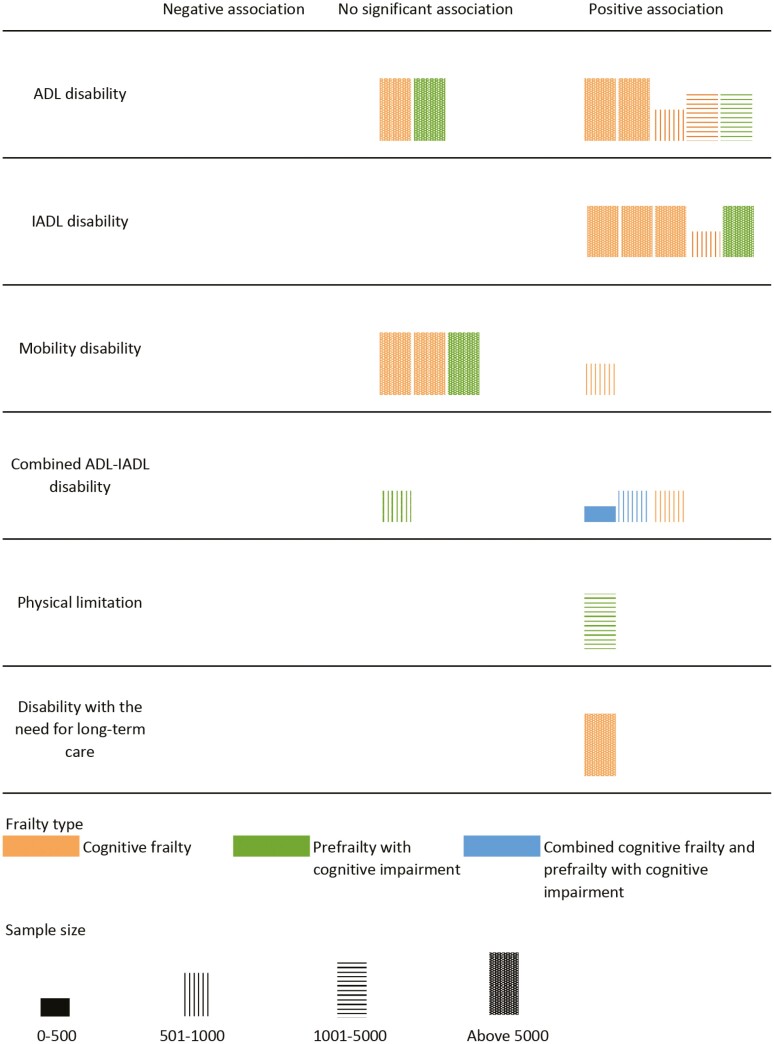
Harvest plot of the association between cognitive frailty types and functional disability. *Notes*. ADL = activities of daily living; IADL = instrumental activities of daily living. Combined ADL–IADL disability was measured using the Katz ADL scale and Lawton and Brody scale, WHO Disability Assessment Schedule, or Groningen Activity Restriction Scale.

Five longitudinal and prospective cohort studies (3,18,31,32, 37) involving 20 778 participants described the relationship between cognitive frailty and ADL disability (Table 1). These studies reported that older adults with cognitive frailty had a higher risk of incident ADL disability than robust older adults (Figure 1). In a national cohort study in the United States (3), participants with cognitive frailty had a higher risk of incident ADL dependency (sub-hazard ratio [sHR]: 2.00; 95% CI: 1.60–2.60) compared with robust participants with normal cognition after 8 years of follow-up. Similar findings were reported in a 4-year longitudinal cohort study in China (18), where cognitively frail participants had a twofold higher risk of developing ADL disability compared to robust participants (odds ratio [OR]: 2.22; 95% CI: 0.97–5.08). Older adults with cognitive frailty were reported to incur a higher burden of ADL disability over 11 years (adjusted rate ratio [aRR]: 20.60; 95% CI: 15.70–26.90) compared to the group without cognitive frailty (37).

**Table 1.** Summary of Primary Outcomes

Comparison Group	ADL Disability		Instrumental ADL Disability		Mobility Disability	
	First author, Publication Year	Statistical Measure (95% CI)	First Author, Publication Year	Statistical measure (95% CI)	First Author, Publication Year	Statistical Measure (95% CI)
Frailty without cognitive impairment (physical frailty) versus robust	Avila-Funes (2009)	OR 2.50 (1.14–5.49)	Avila-Funes (2009)	OR 2.30 (1.49–3.55)	Avila-Funes (2009)	OR 1.32 (0.70–2.52)
	Chen (2020)	OR 2.31 (1.53–3.48)	Chen (2020)	OR 2.23 (1.43–3.47)	Chen (2020)	OR 1.29 (0.52–3.18)
	Ma (2021)	OR 5.47 (2.03–14.75)				
	Aliberti (2019)	sHR 1.70 (1.40–2.00)				
Frailty with cognitive impairment (cognitive frailty) versus robust	Avila-Funes (2009)	OR 5.60 (2.13–14.70)	Avila-Funes (2009)	OR 3.17 (1.47–6.83)	Avila-Funes (2009)	OR 3.88 (0.78–19.41)
	Chen (2020)	OR 2.22 (0.97–5.08)	Chen (2020)	OR 2.80 (1.00–7.87)	Chen (2020)	OR 1.00
	Ma (2021)	OR 10.48 (2.98–36.80)	Shimada (2016)	OR 2.63 (1.74–3.97)	Liu (2018)	aRR 3.10 (2.60–3.60)
	Aliberti (2019)	sHR 2.00 (1.60–2.60)	Liu (2018)	aRR 2.30 (2.10–2.60)		
	Liu (2018)	aRR 20.60 (15.70–26.90)				
Prefrailty without cognitive impairment versus robust	Avila-Funes (2009)	OR 0.72 (0.38–1.36)	Avila-Funes (2009)	OR 1.49 (1.17–1.89)	Avila-Funes (2009)	OR 1.34 (1.13–1.59)
	Ma (2021)	OR 2.34 (1.18–4.63)				
Prefrailty with cognitive impairment versus robust	Avila-Funes (2009)	OR 1.17 (0.45–3.04)	Avila-Funes (2009)	OR 2.83 (1.91–4.19)	Avila-Funes (2009)	OR 1.15 (0.76–1.73)
	Ma (2021)	OR 3.28 (1.31–8.23)				

*Notes*: ADL = activities of daily living; aRR = adjusted rate ratio; OR = odds ratio; sHR = sub-hazard ratio.

#### IADL disability

The association between cognitive frailty and IADL disability was reported in 3 longitudinal/prospective cohort studies and 1 cross-sectional study (18,31,37,39) encompassing 20 697 older adults (Table 1). Similar to ADL, older adults with cognitive frailty had a higher incidence and burden of IADL disability (Figure 1). Two longitudinal cohort studies in France and China showed that cognitively frail individuals were at increased risk of IADL disability compared to robust individuals (OR: 3.17; 95% CI: 1.47–6.83 in (31) and OR: 2.80; 95% CI: 1.00–7.87 in (18), respectively). Similarly, older adults with prefrailty and cognitive impairment had a higher IADL disability risk, with this association being significant albeit weaker than cognitive frailty (31). In a cross-sectional study involving 8 864 Japanese older adults from the National Center for Geriatrics and Gerontology–Study of Geriatric Syndromes cohort (39), cognitive frailty was associated with IADL limitation (OR: 2.63; 95% CI: 1.74–3.97).

#### Mobility disability

There was very limited evidence of the association between cognitive frailty and mobility disability (Table 1). Nevertheless, the burden of mobility disability was reported to be substantially higher in participants with cognitive frailty than those without (aRR: 3.10; 95% CI: 2.60–3.60) (37).

### Other Outcomes

A longitudinal cohort study in China (36) showed that the odds of incident physical function limitation were increased in individuals with prefrailty and cognitive impairment or prefrailty alone compared with robust individuals after 4 years of follow-up, but this was higher in the prefrailty and cognitive impairment group (OR: 1.78; 95% CI: 1.26–2.51). In 2 studies that combined cognitive frailty and prefrailty and reported disability outcomes using different measures (33,35), the risk of disability was at least 4 or 5 times higher in the combined cognitive frailty group compared with the robust group. The risk of incident disability based on the need for long-term care certification (LTCI) in Japan was higher among participants with cognitive frailty compared with robust participants (hazard ratio [HR]: 3.86; 95% CI: 2.95–5.05) (34). The mandatory social LTCI system in Japan categorizes older adults according to levels of need based on monthly follow-up for 24 months, and the onset of long-term care/support needs in movement, ADL, IADL, and other functions denotes incident disability.

A cross-sectional study in Italy revealed that cognitively frail individuals were more likely to develop ADL–IADL disability than robust individuals, but no differences in the association with disability were observed between prefrailty with cognitive impairment and robust groups (38).

## Discussion and Implications

In this review, we found 11 studies that examined ADL, IADL, or mobility disability among older adults with cognitive frailty. We observed that the measurement of cognitive frailty and prefrailty varied, resulting in the mixed prevalence of cognitive frailty in the community setting. Across all studies, community-dwelling participants with cognitive frailty were reported to have an increased risk of functional disability, particularly in ADL and IADL, compared with robust participants with normal cognition.

Findings are in line with previous reviews on cognitive frailty (16,17), where cognitive frailty was predictive for adverse outcomes, including functional disability. However, due to the limited number of cohort studies that reported on the use of similar statistical effects or outcome measurements, evidence remains limited for robust comparisons for functional disability outcomes in cognitive frailty. Our review identified 4 additional studies that reported the association between cognitive frailty and functional disability, but similar limitations hindered a pooled analysis of findings. There were another 4 studies that reported the association between cognitive frailty and functional disability but did not meet the inclusion criteria (age (4), population characteristics (28,40), and outcome combined with mortality (41)). Although there is an agreement that cognitive frailty is associated with an increased risk of ADL or IADL disability in these studies, the use of standardized cognitive frailty or prefrailty definition and measurements would better facilitate comparisons of frailty subtypes and the risk of disability. Previous studies have similarly reported differences in predictive values on adverse outcomes depending on the operationalization of frailty/phenotype criteria or cognitive function assessments (14,16,30,42).

The association between cognitive frailty and functional disability was stronger than physical frailty in 7 of 9 longitudinal cohort studies included in this review. Nevertheless, studies to date do not allow for a comparison that could show the higher importance of assessing cognitive frailty over physical frailty or cognitive impairment alone for this adverse outcome. Previous studies have suggested that the higher risk of adverse outcomes in cognitive frailty is attributed to the cumulative risk of adverse outcomes conferred when physical frailty and cognitive impairment coexisted (16,18). This led to the suggestion for the inclusion of cognitive function in frailty assessment to improve the identification of adverse outcomes among frail older adults (3,4,16,36). Despite this, the synergistic interaction of frailty/prefrailty and cognitive impairment on adverse health outcomes is still not well understood (17), necessitating further research in this area. This may be important in evaluating functional disability as an adverse outcome, given that frailty or cognitive impairment alone has been associated with an increased risk of functional disability (15,21,43–45).

Among prefrail older adults with cognitive impairment, results indicated an increased odds of developing ADL or IADL disability compared with robust older adults, but associations with the risk of disability were much weaker compared to cognitive frailty in studies that reported on both frailty and prefrailty. The presence of cognitive impairment did not appear to be a significant predictor of disability among those with prefrailty. As only a few studies reported on prefrailty with cognitive impairment, and its association with primary and other outcomes (31–33), we urge caution in the interpretation of review findings for this group. As such, more research is needed to understand the association between prefrailty with cognitive impairment and adverse outcomes, given that early prefrailty or cognitive impairment may be reversible with targeted interventions, thus allowing the prevention of transition to frailty and development of functional disability (9,36).

Only a few included studies reported on mobility disability or physical function limitation among older adults with cognitive frailty. Within the literature, studies have focused on mobility decline or disability as a risk factor for frailty (46–49), which may explain the lack of reporting of mobility disability as an adverse outcome of cognitive frailty. Further research will be needed to ascertain the association between cognitive frailty and mobility disability. The assessments for mobility disability or functional disabilities other than ADL or IADL disability were also varied, which limits comparisons across populations.

This study has several limitations. Studies included in the review were heterogeneous due to differences in inclusion and exclusion criteria, participant characteristics, frailty (modified phenotype criteria), and cognitive function assessments. We included observational studies, where a causal relationship between cognitive frailty and outcomes cannot be ascertained. Hospitalized and institutionalized participants are more vulnerable to adverse health outcomes irrespective of frailty status (50) and, thus, were not included in this review, but this might limit the generalizability of the findings. We did not include non-English language articles and, therefore, might have missed related studies.

Differences in cognitive frailty prevalence were observed due to differences in the study population and cognitive frailty classifications. Future research should focus on these gaps to estimate cognitive frailty burden and inform health and social care policy, especially for lower–middle- and low-income countries. With an increased risk of functional disability, early prevention of cognitive frailty or its progression through screening of frailty and cognitive impairment is vital. The effort to incorporate this into routine health care of older adults is needed (10), given that screening is often hindered by impracticability and not widely practiced in primary care. Above all, more longitudinal cohort studies and consensus on assessing and classifying cognitive frailty/prefrailty with cognitive impairment (17) would allow better comparisons across populations.

The present review found that older adults with cognitive frailty were likely to have an increased risk of developing disabilities than robust older adults. Further research is needed to ascertain the associations between prefrailty with cognitive impairment and the risk of disability. With functional disability and frailty being emerging public health issues, further research on cognitive frailty, especially in rapidly aging populations, is needed to advance our understanding of the burden of this condition, prevention of its adverse outcomes, and maintenance of functional ability among older adults.

## Supplementary Material

igad005_suppl_Supplementary_Material

## Data Availability

All data relevant to the study are included in the article or uploaded as supplementary information.
