# Supplementary material for: Cognitive Frailty and Functional Disability Among Community-Dwelling Older Adults: A Systematic Review
Source: Innov Aging. 2023 Jan 23;7(2):igad005. doi: 10.1093/geroni/igad005 (PMC9999676; doi:10.1093/geroni/igad005)
Supplement: igad005_suppl_Supplementary_Material [file igad005_suppl_supplementary_material.docx]

*Innovation in Aging* Online Supplementary Material: Kar Foong Tang, Pei-Lee Teh, & Shaun Wen Huey Lee. Cognitive frailty and functional disability among community-dwelling older adults: A systematic review.

Text S1 Search terms and databases.

The following search terms were used in PubMed from January 2001 to 14 May 2022. Search terms were adjusted in Embase, CINAHL Plus and PsycINFO.

**Search terms**

1. frail OR frailty
2. older OR elderly OR old OR senior
3. mobility OR movement
4. physical function OR activities of daily living OR activity capacity OR functional capacity OR disability
5. (adverse OR negative) outcome
6. 1 AND 2
7. 3 OR 4 OR 5
8. 6 AND 7


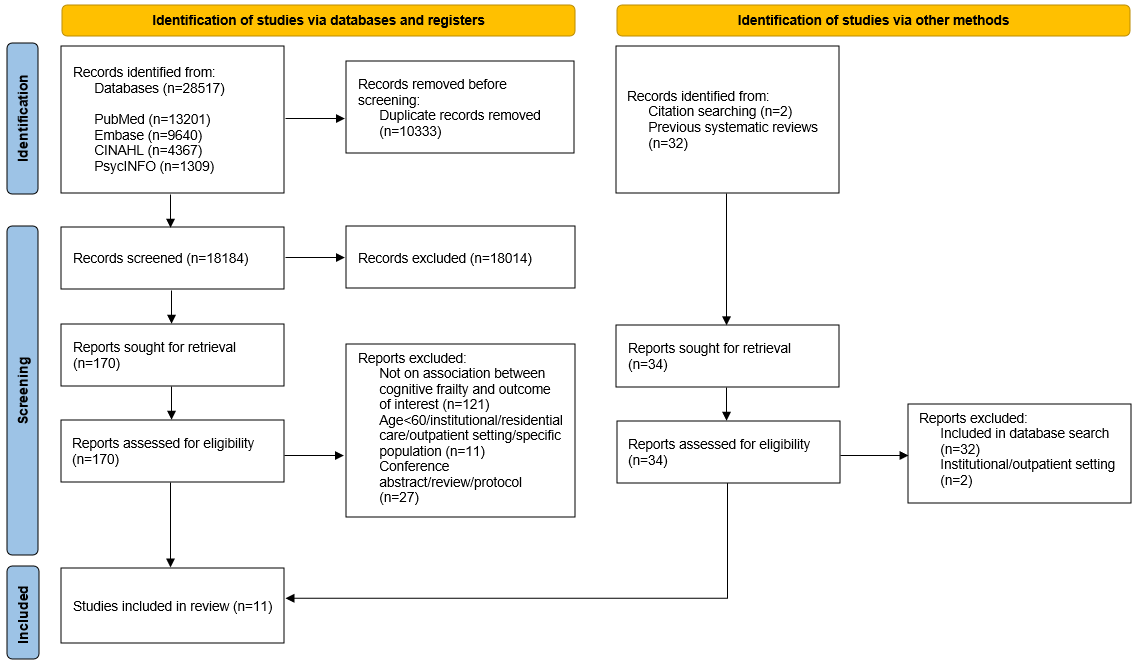


Figure S1. Study search flow.

Table S1. Characteristics of included studies.

| **First author, publication year** | **Study design** | **Cohort** | **Country** | **Participants (N)** | **Female participants (%)** | **Age in years (Range)** | **Age in years, (Mean [SD])** | **Follow-up duration (year)** | **Frailty assessment** | **Frailty types or classification** | **Cognitive assessment** | **Cognitive score classification** |
| --- | --- | --- | --- | --- | --- | --- | --- | --- | --- | --- | --- | --- |
| Aliberti (2019) | Prospective cohort study | Health and Retirement Study (HRS) | United States | 7338 | 55.8 | 65 years and older | 74.4 (7.0) | 8 | Fried frailty phenotype | Individuals who met three or more components were classified as physically frail. Participant group: (1) no deficit, cognitively normal and non-physically frail; (2) only cognitively impaired; (3) only physically frail; and (4) cognitively impaired and physically frail. | (1) immediate and delayed recall of 10 common nouns, (2) serial subtractions by 7, and (3) a backward count task from 20. | The sum of the three tests results in a 27-point scale, with higher scores indicating better cognitive functioning. The 27-point scale classified individuals as having normal cognition (scores 12–27) and cognitive impairment without dementia (CIND) (scores 7–11). Scores lower than seven indicated dementia. |
| Avila-Funes (2009) | Longitudinal cohort study | Three-City Study (3C) | France | 6030 | 61.2 | 65 to 95 | 74.1 (5.2) | 4 | Fried frailty phenotype | Subjects meeting three or more criteria were classified as frail, those meeting one or two as prefrail, and those meeting none as non-frail.  Participant groups: non-frail without CoI, non-frail with CoI, prefrail without CoI, prefrail with CoI, frail without CoI, and frail with CoI. | MMSE, The Isaacs Set Test | Four levels of cognitive function were defined according to the quartiles of scores distribution. The lowest quartile of the distribution indicates the worst performance. Subjects in the lowest quartile in both tests were considered cognitively impaired. |
| Chen (2020) | Longitudinal cohort study | China Health and Retirement Longitudinal Study (CHARLS) | China | 5113 | 48.5 | 60 years and older | 68.1 (6.5) | 4 | Fried frailty phenotype, adapted version | Participants who met three or more criteria were defined as having physical frailty (frail); otherwise, they were considered to have no physical frailty (non-frail), and those with CoI and frail were considered to have CF. | Telephone Interview of Cognitive Status (TICS-10), word recall, and figure drawing | Overall cognition score is the sum score of the TICS-10 (orientation and attention, 0-10), word recall (episodic memory, 0-10), and figure drawing (visual-spatial abilities, 0-1). Participants were classified as having CoI if their summary score fell more than 1 SD below age-appropriate norms; otherwise, they were defined as having normal cognition |
| Liu (2018) | Longitudinal study data analysis | Yale Precipitating Events Project (PEP) | United States | 690 | 65.4 | 70 years and older | NA | 11 | Modified Fried frailty phenotype | Four distinct joint trajectories were identified as no CF, slow cognitive decline and progressive frailty, rapid cognitive decline and progressive frailty, and CF. | MMSE | Higher scores indicate better performance (range: 0-30) |
| Ma (2021) | Retrospective analysis of a prospective cohort study | Rugao Longevity and Aging Study (RuLAS) | China | 1607 | 52.3 | 70–84 | 75.2 (3.9) | 3 | Modified Fried frailty phenotype | For physical frailty, those with three or more criteria were classified as frailty, one or two as pre-frailty, and no criteria present was classified as robust. Participants were divided into six groups according to physical frailty and cognitive function: (1) robust without CoI, (2) pre-frail without CoI, (3) frail without CoI, (4) robust with CoI, (5) pre-frail with CoI, and (6) frail with CoI. CF was defined as frail with CoI, and others were defined as non-CF. | Revised Hasegawa dementia scale (HDS-R) | The total possible score is 32.5. According to the definition of CF, those with severe CoI (HDS-R ≤ 10) were excluded. The lowest 20% of the HDS-R scores was defined as CoI. The lowest 20% of the HDS-R scores after stratification by age and education level were defined as lower cognitive performance (illiterate participants aged 70–79 ≤ 14.5 points, literate participants aged 70– 79 ≤ 20.5 points, illiterate participants aged 80– 84 ≤ 13.5 points, and literate participants aged 80– 84 ≤ 18.0 points) |
| Rivan (2021) | Prospective cohort study | Longitudinal Study on Neuroprotective Model for Healthy Longevity (LRGS TUA) | Malaysia | 400 | 54.9 | 60 years and above | 67.65 (5.26) | 5 | Fried frailty phenotype | Physical frailty was assessed using Fried et al. criteria and the cut-off points outlined in the CHS. CF was categorised based on the presence of both physical (pre-frailty/frailty) and cognitive (subjective cognitive complaint/MCI). | Global cognitive function was assessed using the Malay version of MMSE (M-MMSE) and the Montreal Cognitive Assessment; the Weschler Memory Scale-Revised and the Digit Span Forward and Backward test was used to assess attention and working memory; the Digit Symbol test was administered to measure information processing speed; and RAVLT for verbal learning and memory. | Participants were categorised as having a MCI if they had subjective memory complaint (either by participants or caregivers), objective memory impairment (at least 1.5 SD below the mean for either RAVLT or digit span tests), without or very minimal functional limitations in basic ADL (at least 1.5 SD below the mean), maintained global cognitive function (M-MMSE score of > 19) and not diagnosed to have dementia, reported by a doctor/physician at baseline. |
| Roppolo (2017) | Cross-sectional study | NA | Italy | 594 | 64.0 | 65 to 90 | 73.6 (5.8) | No follow-up (cross-sectional study) | Fried frailty phenotype, adapted version | Participants who scored positive to three or more criteria were classified as frail, those who scored positive to one or two as pre-frail, and those meeting none as robust. | MMSE | The score ranges from 0 to 30. Higher scores indicate a better level of cognitive functioning. To distinguish between those with high and low cognitive functioning, a cut-off equal to or less than 25 was used. |
| Shimada (2016) | Cross-sectional study (Data from prospective cohort study) | National Center for Geriatrics and Gerontology–Study of Geriatric Syndromes (NCGG-SGS) | Japan | 8864 | 52.0 | 65 years and older | 73.5 (5.3) (participants without IADL limitation at baseline) | No follow-up (cross-sectional study) | Fried frailty phenotype | Participants with none of the components were considered non-frail, and those with one or two components were considered to be pre-frail. Those with three or more components were considered to be frail.  Participant group: 1) robust older individuals who had no physical frailty and CoI (robust group), 2) physically frail older adults without CoI (physical frailty group), 3) non-physically frail older adults with major CoI (CoI group), and 4) physically frail older adults with CoI (CF group). | NCGG-FAT | Score <1.5 SDs below the age and education-specific means for a population-based cohort of community-dwelling older adults. Major CoI is characterised by deficits on two or more of the tests in the NCGG-FAT. Participants without deficits on these tests were considered cognitively intact. |
| Tsutsumimoto (2020) | Prospective cohort study | National Center for Geriatrics and Gerontology–Study of Geriatric Syndromes (NCGG-SGS) | Japan | 9936 | 51.7 | 65 years and older | 73.5 (5.4) | 2 | Walking speed and grip-strength measurements | Participants with either of these two physical deficits were considered physically frail.  Four groups: robust (non-physically frail older adults without CoI), CoI (non-physically frail older adults with CoI), physical frailty (physically frail older adults without CoI, CF (physically frail older adults with CoI). | NCGG-FAT | All tests used had established standardised thresholds for the definition of impairment in the corresponding domain (i.e., a score <1.5 SDs below the age and education-specific means) for a population-based cohort of community-dwelling older adults. We determined the presence of CoI among participants who fell under the standardised threshold in one or more NCGG-FAT tests. |
| Wang (2021) | Prospective cohort study | NA | China | 735 | 67.9 | 60 years or older | 68.9 (6.3) | 1 | Fried frailty phenotype | For Fried's phenotype, a score of 0 represents robustness, 1 to 2: pre-physical frailty, and 3: physical frailty. Four categories based on the two components-physical frailty and cognitive status measurements: Category 1: Robustness and normal cognition; Category 2: Robustness and SCD; Category 3: Pre-physical frailty or physical frailty and normal cognition; Category 4: Pre-physical frailty or physical frailty and SCD (reversible CF). | The identification of SCD was based on the criteria of the SCD-I Working Group. | (1) self-experienced persistent decline in cognitive capacity in comparison with previously normal status and unrelated to an acute event, (2) normal age-, gender- and education-adjusted performance on standardised cognitive tests which are used to classify MCI or prodromal AD. Participants recognised as having intact cognitive function by the SPMSQ were further assessed with a simplified SCD questionnaire to identify SCD, including four self-report cognitive domains: memory, naming, orientation, and mathematical reasoning. SCD was diagnosed if a positive response was given to one of the four cognitive domains |
| Yu (2018) | Prospective cohort study | Osteoporotic Fractures in Men (MrOs) and Women (MsOs) (Hong Kong) Study | China (Hong Kong) | 3491 | 48.4 | 65 years and older | 72.03 (4.91) | 4 (for physical limitation outcome) | Fried frailty phenotype | Robust (score=0), pre-frail (score=1–2), and frail (score=3–5). Participant group: (1) robust and cognitively intact, (2) robust and cognitively impaired, (3) pre-frail and cognitively intact, and (4) pre-frail and cognitively impaired. | Cantonese version of MMSE (CMMSE) | A score of less than 21 in individuals with no education, less than 24 in individuals with primary education, or less than 27 in well-educated individuals with secondary or tertiary education is identified as having CoI. Alternatively, individuals who failed to recall any of the three words during the CMMSE delayed recall (i.e., a CMMSE delayed recall score of less than 3) or were unable to complete one or more language and praxis tasks on the CMMSE (i.e., a CMMSE language and praxis score of less than 9) were classified as having CoI. |

*Note.* ADL: activities of daily living, CF: cognitive frailty, CHS: Cardiovascular Health Study, CoI: cognitive impairment, SCD: subjective cognitive decline, IADL: instrumental activities of daily living, MMSE: Mini-Mental State Examination, MCI: mild cognitive impairment, NCGG-FAT: National Center for Geriatrics and Gerontology-Functional Assessment Tool.

Table S2. Prevalence by frailty types and functional disability measures.

| **First author, publication year** | **Prevalence of cognitive frailty (%)** | **Prevalence of prefrailty with cognitive impairment (%)** | **Adverse outcome** | **Adverse outcome measure** | **Covariate adjustment** | **Analysis** |
| --- | --- | --- | --- | --- | --- | --- |
| Aliberti (2019) | 5.0 | n/a | Incident ADL dependence | Participants’ need for ADL help was assessed during follow-up waves every two years. Participants were classified as having incident ADL dependence if they reported needing help in any of these six daily activities: eating, transferring, walking across the room, dressing, toileting, and bathing. Because the exact date of incident ADL disability was unavailable, the date of the event was considered as the median time between the two waves. | Age, sex, ethnicity, education, net worth, marital status, comorbidities (stroke, hypertension, diabetes, cancer, lung disease, heart disease, and depression), and smoking status. | Competing risk hazard models |
| Avila-Funes (2009) | 1.5 | 5.7 | ADL, IADL, mobility disability | Mobility was assessed according to the Rosow-Breslau scale (Guttman’s health scale): doing heavy housework, walking half a mile, and going up the stairs. For IADL, participants indicated whether they were able to perform eight IADLs based on the Lawton–Brody scale, such as using a telephone, being responsible for their medication, managing money, and ability to use public or private transport, shopping, grooming, doing housework, and doing laundry. For ADL, participants were asked whether they needed help with any task on the Katz ADL scale (bathing, dressing, transferring from bed to chair, toileting, and feeding). For each domain of disability, if participants indicated that they could not perform one or more activities without help, they were considered as having mobility, IADL, or ADL disability. The first episode of disability was considered, and a 4-year incident disability was established only in subjects without prevalent disability in the same domain at baseline. | Age, sex, education level, income, smoking status, drinking status, number of chronic diseases, self-reported health and Center for Epidemiologic Studies Depression Scale score (excluding the two questions used for the frailty definition). For incident IADL disability, ORs were adjusted for baseline mobility disability. For incident ADL disability, ORs were adjusted for baseline mobility and IADL disability. | Logistic regression |
| Chen (2020) | 1.6 | n/a | BADL, IADL, mobility disability | 5-BADL (dressing, bathing, eating, getting in/out of bed, and using the toilet), 5-IADL (managing money, taking medications, shopping for groceries, meal preparation, and cleaning house), and 7-item mobility activities (100 metres, climbing several flights of stairs, getting up from a chair, stooping or kneeling or crouching, extending arms up, lifting 11 lb, and picking up a small coin) were assessed. For each task in the three domains, participants were asked, “Do you have difficulty performing the task?”. Participants who needed personal assistance performing one or more of the corresponding activities in each domain were defined as having a disability. Since the timing of developing disability during the follow-up period was not available, a binary outcome was defined to denote the occurrence of disability over the 4-year follow-up. | Age, gender, residence, education, marital status, smoking status, alcohol consumption, BMI, and multimorbidity. | Logistic regression |
| Liu (2018) | 6.5 | n/a | ADL, IADL, mobility disability | Participants were asked, “At present, do you need help from another person to (complete the task)?” for each of 4 basic ADLs (bathing, walking, dressing, and transferring), 5 IADLs (shopping, housework, meal preparation, taking medications, and managing finances), and three mobility activities (walk one-quarter mile, climb a flight of stairs, and lift/carry 10 lb). Disability in 3 functional domains (ADL, IADL, and mobility) was operationalised as the need for personal assistance in performing one or more of the corresponding activities. | Age, sex, race, education, living alone, number of chronic conditions, and depression symptom. | Joint trajectory modelling approach, Poisson models (generalised estimating equations) |
| Ma (2021) | 2.9 | n/a | ADL disability | Disability was measured using the ADL scale. The incident of ADL disability was defined as one difficulty in any ADL (bathing, using the toilet, dressing, transferring, and eating) after three years but no disability at baseline. | Age, sex, educational level, self-assessment of health status at baseline, baseline diagnosed depression, occupation, baseline marital status, baseline tobacco consumption, baseline intake of alcohol, baseline BMI, baseline diagnosed diabetes, and baseline diagnosed hypertension. | Logistic regression |
| Rivan (2021) | 39.3^a^ | n/a | Disability (self-care, life activities, mobility, participation) | Disability was assessed using the WHODAS, which captures six major domains: self-care, participation, cognition, mobility, getting along, and life activities. WHODAS 2.0, a 12-item questionnaire with a five-point Likert scale (0: none,1: some, 2: moderate, 3: severe and 4: very severe), assesses restriction in activities of daily living and social participation due to health problems for the past month. Levels of disability were categorised as 0 = no disability, 1 to 4 = mild disability, 5 to 9 = moderate disability, and 10 to 48 + more severe disability. Participants were grouped into two groups, severe disability and without disability. | Age, years of education, waist circumference, chair stand test, back scratch test, social support, depression. | Logistic regression |
| Roppolo (2017) | 4.4 | n/a | Disability (ADL and IADL combined) | Disability was rated using the Groningen Activity Restriction Scale, composed of 18 items about autonomy in the performance of basic and instrumental ADL. Each item has four categories of response, with a total score ranging from 18 (absence of disability) to 72 (severe disability). | Age and gender. | Two-way analysis of covariance (ANCOVA) |
| Shimada (2016) | 1.4 | n/a | Ability to perform IADL | The items of IADL were based on the Lawton and Brody IADL scale. Subjects were asked whether they had performed each activity during the past month; responses were ‘yes (did)’ or ‘no (did not)’. The number of activities for which each participant answered ‘yes’ for IADL was added, and individuals who answered ‘‘no’’ in one or more of the four IADL items were defined as having an IADL limitation. | Two demographic variables (age and sex), three physiological variables, four primary diseases or geriatric syndromes, and six psychosocial variables. | Logistic regression |
| Tsutsumimoto (2020) | 11.2 | n/a | Incident disability (needing LTCI) | Participants were followed up monthly to determine incident disability according to the LTCI system for 24 months after the baseline assessment. The mandatory social LTCI system categorises older adults according to seven levels of need. Questionnaires are administered to determine movement, balance, ADL, IADL, cognition, and special assistance requirements. The onset of long-term care/support needs is the point when the patient is certified for LTCI. | Age, sex, education, BMI, medication, hypertension, hyperlipidemia, diabetes, stroke, osteoarthrosis, current drinking habit, current smoking habit, physical inactivity | Cox proportional hazard model |
| Wang (2021) | 27.8^a^ | n/a | ADL-IADL disability | The Katz index was used to assess ADL, and the Lawton scale was used to assess IADL. Participants with one or more difficulties on the Katz index or the Lawton scale were defined as having ADL-IADL disability. Adverse outcomes were defined as adverse events that did not occur at baseline but at 1-year follow-up. | Age, gender, education, monthly income, marital status, comorbidity, social support score, current smoking, drinking, baseline ADL-IADL disability (excluded for disability as outcome). | Logistic regression |
| Yu (2018) | n/a | 8.7 | Incident physical limitation | Physical limitation was assessed using two questions: do you have any difficulty in climbing stairs (answers: no, a little, a lot) and do you have any difficulty in carrying out the following household activities such as moving chairs or tables (answers: no, a little, a lot). Participants were categorised as having physical limitations if the answer to either question was “a little” or “a lot,” while those who answered “no” to both questions were categorised as having no physical limitations. Incident physical limitation was defined as a progression from those without limitation at baseline to having limitation at follow-up. | Age, sex, educational level, socioeconomic status, smoking habit, alcohol intake, physical activity, Diet Quality Index-International, BMI, and baseline values of the respective outcome variable. | Logistic regression |

*Note*. ^a^ Combined prevalence of cognitive frailty and cognitive prefrailty. ADL: activities of daily living, BMI: body mass index, IADL: instrumental activities of daily living, n/a: not available, OR: odds ratio, LTCI: long-term care insurance, RAVLT: Rey Auditory Verbal Learning Test; WHODAS: WHO Disability Assessment Schedule.

Table S3. Quality assessment.

The Joanna Briggs Institute’s Critical Appraisal Checklist for cohort studies

| First author, publication year | 1. Were the two groups similar and recruited from the same population? | 2. Were the exposures measured similarly to assign people to both exposed and unexposed groups? | 3. Was the exposure measured in a valid and reliable way? | 4. Were confounding factors identified? | 5. Were strategies to deal with confounding factors stated? | 6. Were the groups/participants free of the outcome at the start of the study (or at the moment of exposure)? | 7. Were the outcomes measured in a valid and reliable way? | 8. Was the follow-up time reported and sufficient to be long enough for outcomes to occur? | 9. Was follow-up complete, and if not, were the reasons for loss to follow-up described and explored? | 10. Were strategies to address incomplete follow-up utilised? | 11. Was appropriate statistical analysis used? | Overall appraisal |
| --- | --- | --- | --- | --- | --- | --- | --- | --- | --- | --- | --- | --- |
| Aliberti (2019) | Yes | Yes | Yes | Yes | Yes | Yes | Unclear | Yes | Yes | N/A | Yes | Include |
| Avila-Funes (2009) | Yes | Yes | Yes | Yes | Yes | Yes | Unclear | Yes | Yes | N/A | Yes | Include |
| Chen (2020) | Yes | Yes | Yes | Yes | Yes | Yes | Unclear | Yes | Yes | N/A | Yes | Include |
| Liu (2018) | Yes | Yes | Yes | Yes | Yes | Yes | Unclear | Yes | Yes | N/A | Yes | Include |
| Ma (2021) | Yes | Yes | Yes | Yes | Yes | Yes | Unclear | Yes | Yes | N/A | Yes | Include |
| Rivan (2021) | Yes | Yes | Yes | Yes | Yes | Yes | Unclear | Yes | Yes | N/A | Yes | Include |
| Tsutsumimoto (2020) | Yes | Yes | Yes | Yes | Yes | Yes | Yes | No | Yes | N/A | Yes | Include |
| Wang (2021) | Yes | Yes | Yes | Yes | Yes | Yes | Unclear | No | Yes | N/A | Unclear | Include |
| Yu (2018) | Yes | Yes | Yes | Yes | Yes | Yes | Unclear | Yes | Yes | N/A | Yes | Include |

The Joanna Briggs Institute’s Critical Appraisal Checklist for cross-sectional studies

| First author, publication year | 1. Were the criteria for inclusion in the sample clearly defined? | 2. Were the study subjects and the setting described in detail? | 3. Was the exposure measured in a valid and reliable way? | 4. Were objective, standard criteria used for measurement of the condition? | 5. Were confounding factors identified? | 6. Were strategies to deal with confounding factors stated? | 7. Were the outcomes measured in a valid and reliable way? | 8. Was appropriate statistical analysis used? | Overall appraisal |
| --- | --- | --- | --- | --- | --- | --- | --- | --- | --- |
| Roppolo (2017) | Yes | Yes | Yes | Yes | Yes | Yes | Unclear | Unclear | Include |
| Shimada (2016) | Yes | Yes | Yes | Yes | Yes | Yes | Unclear | Yes | Include |

Table S4. PRISMA (Preferred Reporting Items for Systematic Reviews and Meta-Analyses) checklist.

| **Section and Topic** | **Item #** | **Checklist item** | **Location where item is reported (manuscript page/file)** |
| --- | --- | --- | --- |
| **TITLE** | | |  |
| Title | 1 | Identify the report as a systematic review. | Title page, abstract |
| **ABSTRACT** | | |  |
| Abstract | 2 | See the PRISMA 2020 for Abstracts checklist. | Abstract |
| **INTRODUCTION** | | |  |
| Rationale | 3 | Describe the rationale for the review in the context of existing knowledge. | Introduction |
| Objectives | 4 | Provide an explicit statement of the objective(s) or question(s) the review addresses. | Introduction |
| **METHODS** | | |  |
| Eligibility criteria | 5 | Specify the inclusion and exclusion criteria for the review and how studies were grouped for the syntheses. | Methods |
| Information sources | 6 | Specify all databases, registers, websites, organisations, reference lists, and other sources searched or consulted to identify studies. Specify the date when each source was last searched or consulted. | Methods |
| Search strategy | 7 | Present the full search strategies for all databases, registers and websites, including any filters and limits used. | Supplemental file |
| Selection process | 8 | Specify the methods used to decide whether a study met the inclusion criteria of the review, including how many reviewers screened each record and each report retrieved, whether they worked independently, and if applicable, details of automation tools used in the process. | Methods |
| Data collection process | 9 | Specify the methods used to collect data from reports, including how many reviewers collected data from each report, whether they worked independently, any processes for obtaining or confirming data from study investigators, and, if applicable, details of automation tools used in the process. | Methods |
| Data items | 10a | List and define all outcomes for which data were sought. Specify whether all results that were compatible with each outcome domain in each study were sought (e.g. for all measures, time points, analyses), and if not, the methods used to decide which results to collect. | Methods |
|  | 10b | List and define all other variables for which data were sought (e.g. participant and intervention characteristics, funding sources). Describe any assumptions made about any missing or unclear information. | Methods |
| Study risk of bias assessment | 11 | Specify the methods used to assess risk of bias in the included studies, including details of the tool(s) used, how many reviewers assessed each study and whether they worked independently, and if applicable, details of automation tools used in the process. | Methods |
| Effect measures | 12 | Specify for each outcome the effect measure(s) (e.g. risk ratio, mean difference) used in the synthesis or presentation of results. | Methods |
| Synthesis methods | 13a | Describe the processes used to decide which studies were eligible for each synthesis (e.g. tabulating the study intervention characteristics and comparing against the planned groups for each synthesis (item #5)). | Methods |
|  | 13b | Describe any methods required to prepare the data for presentation or synthesis, such as handling of missing summary statistics or data conversions. | Not applicable |
|  | 13c | Describe any methods used to tabulate or visually display results of individual studies and syntheses. | Methods |
|  | 13d | Describe any methods used to synthesize results and provide a rationale for the choice(s). If meta-analysis was performed, describe the model(s), method(s) to identify the presence and extent of statistical heterogeneity, and software package(s) used. | Not applicable |
|  | 13e | Describe any methods used to explore possible causes of heterogeneity among study results (e.g. subgroup analysis, meta-regression). | Not applicable |
|  | 13f | Describe any sensitivity analyses conducted to assess robustness of the synthesized results. | Not applicable |
| Reporting bias assessment | 14 | Describe any methods used to assess risk of bias due to missing results in a synthesis (arising from reporting biases). | Not applicable |
| Certainty assessment | 15 | Describe any methods used to assess certainty (or confidence) in the body of evidence for an outcome. | Not applicable |
| **RESULTS** | | |  |
| Study selection | 16a | Describe the results of the search and selection process, from the number of records identified in the search to the number of studies included in the review, ideally using a flow diagram. | Results, Supplemental file |
|  | 16b | Cite studies that might appear to meet the inclusion criteria but which were excluded, and explain why they were excluded. | Discussion |
| Study characteristics | 17 | Cite each included study and present its characteristics. | Results |
| Risk of bias in studies | 18 | Present assessments of risk of bias for each included study. | Results |
| Results of individual studies | 19 | For all outcomes, present, for each study: (a) summary statistics for each group (where appropriate) and (b) an effect estimate and its precision (e.g. confidence/credible interval), ideally using structured tables or plots. | Results |
| Results of syntheses | 20a | For each synthesis, briefly summarise the characteristics and risk of bias among contributing studies. | Results |
|  | 20b | Present results of all statistical syntheses conducted. If meta-analysis was done, present for each the summary estimate and its precision (e.g. confidence/credible interval) and measures of statistical heterogeneity. If comparing groups, describe the direction of the effect. | Not applicable |
|  | 20c | Present results of all investigations of possible causes of heterogeneity among study results. | Not applicable |
|  | 20d | Present results of all sensitivity analyses conducted to assess the robustness of the synthesized results. | Not applicable |
| Reporting biases | 21 | Present assessments of risk of bias due to missing results (arising from reporting biases) for each synthesis assessed. | Not applicable |
| Certainty of evidence | 22 | Present assessments of certainty (or confidence) in the body of evidence for each outcome assessed. | Not applicable |
| **DISCUSSION** | | |  |
| Discussion | 23a | Provide a general interpretation of the results in the context of other evidence. | Discussion |
|  | 23b | Discuss any limitations of the evidence included in the review. | Discussion |
|  | 23c | Discuss any limitations of the review processes used. | Discussion |
|  | 23d | Discuss implications of the results for practice, policy, and future research. | Discussion |
| **OTHER INFORMATION** | | |  |
| Registration and protocol | 24a | Provide registration information for the review, including register name and registration number, or state that the review was not registered. | Abstract, methods |
|  | 24b | Indicate where the review protocol can be accessed or state that a protocol was not prepared. | Methods |
|  | 24c | Describe and explain any amendments to information provided at registration or in the protocol. | Not included |
| Support | 25 | Describe sources of financial or non-financial support for the review and the role of the funders or sponsors in the review. | Funding declaration |
| Competing interests | 26 | Declare any competing interests of review authors. | Conflict of interest declaration |
| Availability of data, code and other materials | 27 | Report which of the following are publicly available and where they can be found: template data collection forms; data extracted from included studies; data used for all analyses; analytic code; any other materials used in the review. | Data sharing statement |

From: Page MJ, McKenzie JE, Bossuyt PM, Boutron I, Hoffmann TC, Mulrow CD, et al. The PRISMA 2020 statement: an updated guideline for reporting systematic reviews. BMJ 2021;372:n71. doi: 10.1136/bmj.n71
